# Enhanced UV-Visible Light Photocatalytic Activity by Constructing Appropriate Heterostructures between Mesopore TiO_2_ Nanospheres and Sn_3_O_4_ Nanoparticles

**DOI:** 10.3390/nano7100336

**Published:** 2017-10-19

**Authors:** Jianling Hu, Jianhai Tu, Xingyang Li, Ziya Wang, Yan Li, Quanshui Li, Fengping Wang

**Affiliations:** Department of Physics, School of Mathematics and Physics, University of Science and Technology Beijing, Beijing 10083, China; jlhu_ustb@163.com (J.H.); jianhaituustb@163.com (J.T.); xl99936@uga.edu (X.L.); wzywolf@163.com (Z.W.); liyan000998@163.com (Y.L.); qsli@ustb.edu.cn (Q.L.)

**Keywords:** photocatalyst, heterostructures, TiO_2_, Sn_3_O_4_

## Abstract

Novel TiO_2_/Sn_3_O_4_ heterostructure photocatalysts were ingeniously synthesized via a scalable two-step method. The impressive photocatalytic abilities of the TiO_2_/Sn_3_O_4_ sphere nanocomposites were validated by the degradation test of methyl orange and •OH trapping photoluminescence experiments under ultraviolet (UV) and visible light irradiation, respectively. Especially under the visible light, the TiO_2_/Sn_3_O_4_ nanocomposites demonstrated a superb photocatalytic activity, with 81.2% of methyl orange (MO) decomposed at 30 min after irradiation, which greatly exceeded that of the P25 (13.4%), TiO_2_ (0.5%) and pure Sn_3_O_4_ (59.1%) nanostructures. This enhanced photocatalytic performance could be attributed to the mesopore induced by the monodispersed TiO_2_ cores that supply sufficient surface areas and accessibility to reactant molecules. This exquisite hetero-architecture facilitates extended UV-visible absorption and efficient photoexcited charge carrier separation.

## 1. Introduction

As a stable, low-cost and environmentally benign material, nanoscaled titanium dioxide (TiO_2_) with unique structural and functional properties has become a widely used semiconductor photocatalyst for various solar-driven clean energy technologies [1]. Tailoring the morphology of TiO_2_ photoanode is a preferred route to achieving high performance in solar cells due to its enhanced properties, such as high surface area, faster electron transport, lower electron-hole recombination rate and good light-harvesting features [2,3]. Nevertheless, the wide optical bandgap of TiO_2_, which seriously limits its light harvesting capability, leaving about 96% of the solar light energy wasted [4]. Compared with the solution of generating donor or acceptor states in the band gap by adding impurities, rationally designing and constructing the surface heterostructures would be a more efficient strategy for achieving an excellent photocatalyst [5].

Recently, Sn_3_O_4_, a novel non-stoichiometric oxide, has raised particular interest in the field of photocatalysis—especially in terms of its catalytic behavior under visible light irradiation—due to a suitable band-gap inside visible light (2.2–2.9 eV) and a distinct surface structure composed of both valences of tin [6,7]. Several studies have shown its great potential as an auspicious photocatalyst under visible light, both for generating hydrogen and degrading dyes [8]. However, some drawbacks have hindered its performance in practice. As a semiconductor with a relatively narrow band gap, pure Sn_3_O_4_ generally leads to a fast recombination of photoexcited electron-hole pairs, which ultimately decreases its degradation rate [9].

Discussing these problems together, it proposes an intriguing idea that Sn_3_O_4_, as the second component, attaches to the surface of TiO_2_ nanostructures, for an exquisite TiO_2_/Sn_3_O_4_ heterostructure. On the one hand, a theoretical analysis indicates that the interface between TiO_2_ and Sn_3_O_4_ is to be a perfect type-II heterojunction (both the potentials of valence band (VB) and conduction band (CB) of Sn_3_O_4_ are higher than that of TiO_2_) [10], which is actually conducive to the separation of photoexcited electron-hole pairs. Furthermore, latest reports have exhibited the superiority of the heterogeneous composite of this kind [11,12]. On the other hand, increased photoactive facets can effectively facilitate the efficiency of photo-absorption and oxygen chemisorption, and bring about a fast rate of surface reactions [13]. Therefore, highly dispersive anatase TiO_2_ mesopore nanospheres, which possess a large number of active surfaces, would likely be an amazing matrix in TiO_2_/Sn_3_O_4_ nanocomposites.

The two-step self-assembly approach is a feasible strategy for the refined design of hierarchical nanostructures with complex morphologies, and has been proven to be an effective way to design multiscale nanostructures, since the morphology and composition obtained from the first step can be further tuned and adjusted by a subsequent second process. Moreover, this approach also allows the combination of multiple synthetic techniques, and the synthesis of complex nanostructures with hierarchical multiscale structures compared with the conventional one-step self-assembling method [14]. Recently, a lot of complex nanostructures with high photocatalytic performance for both visible light and ultraviolet has been acquired by the two-step synthesis method. Usually, these methods can be classified into two categories: (1) synthesis under two continuous identical methods, such as in [9,15,16]; and (2) synthesis under two different methods, such as in [17,18]. In 2015, TiO_2_/Sn_3_O_4_ nanobelts [9] were successfully produced by first synthesizing the TiO_2_ nanobelts, and then assembling Sn_3_O_4_ onto the TiO_2_ nanobelts in a subsequent hydrothermal procedure; in the same year, hierarchical Sn_3_O_4_/N-TiO_2_ nanotubes [17] were synthesized by first weaving N-doped TiO_2_ nanotube via electro-spinning, and then modifying them with Sn_3_O_4_ via hydrothermal reaction. In 2017, a range of heterojunction WO_3_/TiO_2_ thin films were deposited via a two-step process using chemical vapor deposition (CVD) methods [15]. Generally, electro-spinning and chemical vapor deposition is associated with at least one of the following factors: expensive equipment, high voltage, hazardous by-products, or toxic chemicals, rendering the method less environmental friendly and much more complicated than the hydrothermal method or sol-gel synthesis. Hence, synthesizing complex nanostructures via a combination of sol-gel and hydrothermal methods would be a low-cost, scalable, easy to control, and eco-friendly strategy in terms of preparing high-quality, uniform, catalysts.

Herein, we developed a scalable two-step route, combining the sol-gel method and hydrothermal progress to achieve excellent visible and ultraviolet photocatalytic activity by uniformly synthesizing the Sn_3_O_4_ nanoparticles on the surface of TiO_2_ nanospheres. As expected, an enormous enhancement of photocatalytic efficiency was achieved by the distinctive TiO_2_/Sn_3_O_4_ nanocomposites.

## 2. Experimental Section

### 2.1. Chemicals

The chemicals used in this study were of analytic grade, and were used without further purification. Tetrabutyl titanate was purchased from Beijing Xingjin Chemical Factory, Beijing, China. Methyl orange (MO) was obtained from Tianjin Jinke Fine Chemical Industry Research Institute, Tianjin, China. Tin dichloride dihydrate (SnCl_2_∙2H_2_O) and trisodium citrate dihydrate (Na_3_C_6_H_5_O_7_∙2H_2_O) were purchased from Xilong Chemical Industry Co., Ltd., Guangdong, China. Terephthalic acid was purchased from Alfa Aesar (Tianjin, China). All the other organic solvents and salts, including ethylene glycol, acetone, NaOH, were purchased from Sinopharm Chemical Reagent Beijing Co. P25 (nanoscale TiO_2_ powder, surface area 50 m^2^∙g^−1^) was purchased from Degussa AG (Hanau, Germany).

### 2.2. Synthesis of Samples

In this research, we propose a two-step synthesis method to obtain TiO_2_/Sn_3_O_4_ nanocomposites by preparing TiO_2_ core via sol-gel route first and then synthesizing Sn_3_O_4_ on the surface of TiO_2_. (1) Synthesis of core TiO_2_ nanospheres [19]: 3.5 mL tetrabutyl titanate was dissolved in the 50 mL ethylene glycol while stirring vigorously for 10 h. Then, the mixture was immediately poured into a solution containing 170 mL acetone and 2.7 mL deionized water under constant stirring, until white precipitation appeared. The acquired precipitate was calcined in air at 500 °C for 1 h to produce the TiO_2_ powders; (2) Coating TiO_2_ with Sn_3_O_4_: 0.2 g of the TiO_2_ product described above and 5.0 mmol SnCl_2_∙2H_2_O were mixed with 25 mL deionized water, followed by the addition of 12.5 mmol Na_3_C_6_H_5_O_7_∙2H_2_O and 2.5 mmol NaOH under magnetic stirring. During this process, Sn(II) ions were attached to the surface of hydroxyl-rich TiO_2_ spherical colloids through inorganic grafting. The resulting precursor was then transferred to a 50 mL Teflon-lined stainless autoclave and maintained at 180 °C for 12 h. Finally, the collected powder was washed several times with deionized water and ethanol, and dried at 60 °C for 12 h.

### 2.3. Characterization of Samples

In order to obtain the physical and chemical properties of as-prepared samples, several characterizations were conducted. X-ray diffraction (XRD) patterns were recorded by a Rigaku D/MAX-2500 diffractometer with Cu Κα radiation (Rigaku, Tokyo, Japan). Raman spectra were obtained using a HORIBA HR800 spectrometer with an Nd:YAG laser at a wavelength of 532 nm (Horiba Yvon, Paris, France). Scanning electron microscopy (SEM) and transmission electron microscopy (TEM) images were acquired from the ZEISS SUPRA 55 (Zeiss, Oberkochen, Germany)and JEOL JEM-2010 (JEOL, Tokyo, Japan), respectively. X-ray photoelectron spectra (XPS) were recorded on a scanning X-ray microprobe PHI Quantera II (Ulvac-PHI, Chigasaki, Japan). The nitrogen adsorption-desorption isotherm was measured at 77 K on an Autosorb-iQ2-MP analyzer (Quantachrome Instruments, Boynton Beach, FL, USA). The absorption spectra were carried out by UV-visible spectrophotometer (Lambda 950, Perkin-Elmer, Shelton, WA, USA) and the hydroxyl radicals (•OH) trapping photoluminescence spectra were examined by a fluorescence spectrophotometer (Hitachi F-4500, Hitachi, Tokyo, Japan) using excitation a wavelength of 315 nm.

### 2.4. Photocatalytic Experiments

The photocatalytic activity of TiO_2_/Sn_3_O_4_ nanocomposites was evaluated via methyl orange (MO) degradation rate. 80 mL aqueous suspension of MO (20 mg/L) and 80 mg of photocatalyst powder were placed in a 100 mL beaker. Prior to irradiating, the suspensions were magnetically stirred in the dark for 40 min to establish adsorption-desorption equilibrium. A 250 W mercury lamp and a 500 W Halogen lamp with a 420 nm cut-off filter were used as the UV and visible light sources, respectively. After given irradiation time intervals, aliquots of the mixed solution were collected and centrifuged to remove the catalyst particulates for analysis. Four consecutive cycles were tested. The samples were washed thoroughly with water and dried after each cycle.

Using terephthalic acid as a probe molecule, the hydroxyl radicals (•OH) at the photo-illuminated sample/water interface were examined by a special photoluminescence (PL) technique. Terephthalic acid reacts readily with •OH, producing 2-hydroxyterephthalic acid, a great fluorescent material with a unique photoluminescence peak at 426 nm [20], which makes it easy to detect by fluorescence spectrum (excitation wavelength: 315 nm, fluorescence peak: 426 nm). In a typical experiment, 80 mL 0.5 mM terephthalic acid and 2 mM NaOH aqueous solution were completely mixed and then transferred into a 100 mL beaker. The rest of steps are the same as for the degradation of MO.

## 3. Results and Discussion

**Figure 1:**
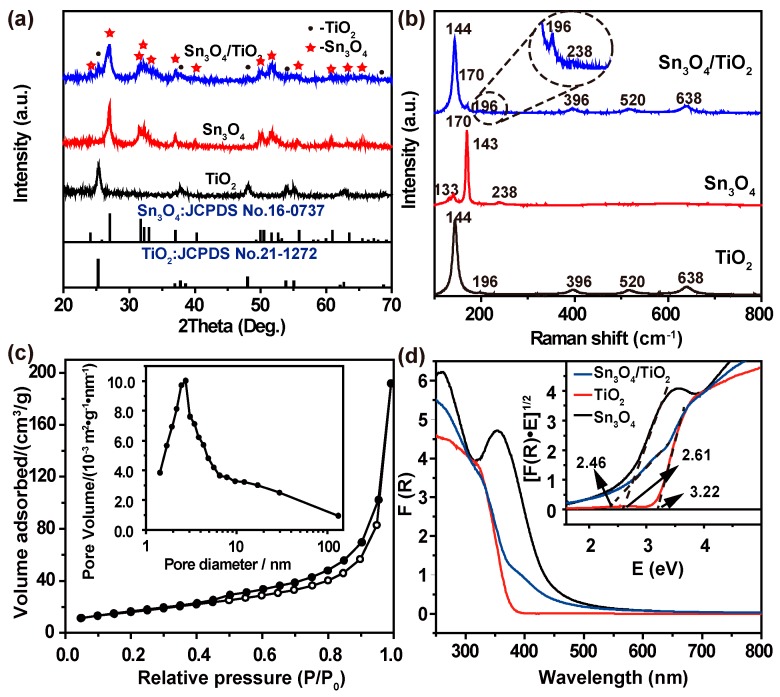
(**a**) XRD patterns, (**b**) Raman spectra of TiO_2_, Sn_3_O_4_ and TiO_2_/Sn_3_O_4_ nanocomposites, (**c**) N_2_ adsorption-desorption isotherms of TiO_2_/Sn_3_O_4_ nanocomposites and (**d**) UV-visible diffuse reflectance spectra and plots of (F(R)*hv*)^1/2^ versus photo energy (right insert) of TiO_2_, Sn_3_O_4_ and TiO_2_/Sn_3_O_4_ nanocomposites.

Figure 1a illustrates the XRD pattern of the TiO_2_/Sn_3_O_4_ nanocomposites. All the diffraction peaks can be indexed to the anatase TiO_2_ (JCPDS 21-1272, marked with black •) and triclinic Sn_3_O_4_ (JCPDS 16-0737, marked with red ☆), validating the high purity of the synthesized TiO_2_/Sn_3_O_4_ composite phase. Compared with the single-phase TiO_2_ and Sn_3_O_4_, all the diffraction peaks are broad and weak, which indicates that the crystallinities are slightly reduced [21]. This result may be attributed to lattice distortion induced by interfacial strain because of different lattice parameters between Sn_3_O_4_ and TiO_2_ [22]. In addition, Raman spectroscopy results (Figure 1b) further confirm the purity of the synthesized TiO_2_/Sn_3_O_4_ composite phase. Specifically, Raman activities of 144, 196, 396, 520 and 638 cm^−1^ were assigned to the anatase TiO_2_ [23], and the 133, 143, 170 and 238 cm^−1^ Raman peaks could be attributed to the Sn_3_O_4_, in accordance with previous reports [7,24]. The textures of the as-synthesized TiO_2_/Sn_3_O_4_, TiO_2_, Sn_3_O_4_ and P25 were characterized by N_2_ physisorption experiments. The Brunauer–Emmett–Teller (BET) surface area data of samples are provided in Table 1. The N_2_ adsorption-desorption isotherm and pore-size distribution of TiO_2_/Sn_3_O_4_ nanocomposites are shown in Figure 1c. The results display that the TiO_2_/Sn_3_O_4_ nanocomposites possess an average pore diameter of 2.733 nm and a larger surface area of 68.1 m^2^/g than the as-prepared TiO_2_ (0.04 m^2^/g), Sn_3_O_4_ (35.2 m^2^/g), and the reported TiO_2_/Sn_3_O_4_ nanobelt heterostructure (51.5 m^2^/g) [9]. Such a high surface-to-volume ratio for the TiO_2_/Sn_3_O_4_ nanocomposites might be of extreme good value in photocatalytic processes, as they would provide more active sites for the adsorption of reactant molecules, and their optical absorbance would increase at visible wavelengths [25]. Figure 1d shows the UV-visible diffusion reflectance spectra (DRS) and plots of (F(R)*hv*)^1/2^ versus photo energy (*hv*) of the TiO_2_/Sn_3_O_4_ along with spectra of the pristine TiO_2_ and Sn_3_O_4_ for comparison. The absorption spectra of the TiO_2_/Sn_3_O_4_ nanocomposites exhibit the mixed absorption properties of both the components. In particular, the absorption edge for TiO_2_/Sn_3_O_4_ nanocomposites is clearly shifted towards visible region (near 505 nm). The optical band gap determined from the plot of the Kubelka-Munk function was found to be 2.46 eV, compared to the observed values of 3.22 eV and 2.61 eV for TiO_2_ and Sn_3_O_4_, respectively. These data reveal the Sn_3_O_4_/TiO_2_ nanocomposites have a lower band gap than the pure Sn_3_O_4_ and TiO_2_ nanoparticles, which is consistent with the published literature [9], and can be explained by the reduced crystallinity of both materials [1], as shown by XRD analysis.

**Figure 2:**
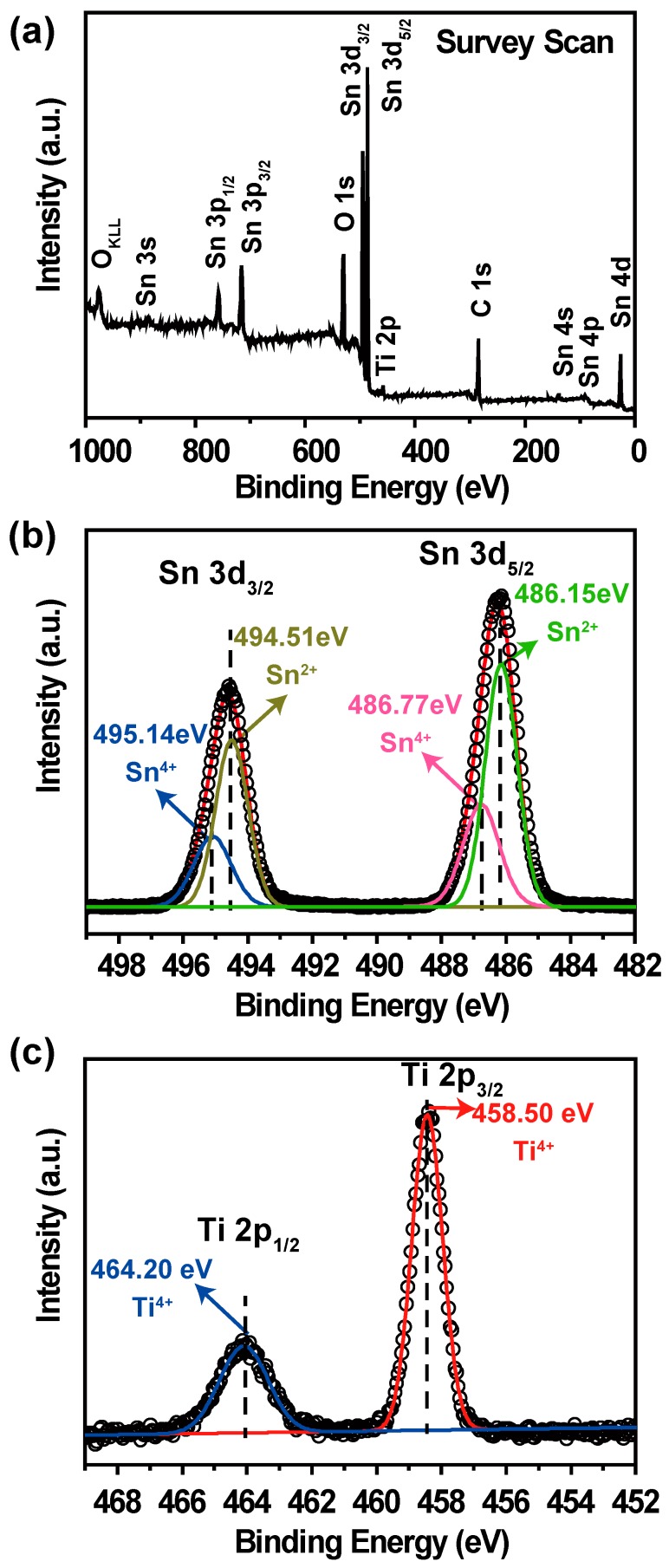
(**a**) Survey scan of XPS, (**b**) Sn 3d core level XPS spectra, and (**c**) Ti 2p core level XPS spectra of the TiO_2_/Sn_3_O_4_ nanocomposites.

The chemical composition and valence state were characterized by X-ray photoelectron spectroscopy (XPS). The full range of XPS spectra, ranging from 0 to 1000 eV, of TiO_2_/Sn_3_O_4_ nanocomposites are shown in Figure 2a. No impurities were observed in the spectra, which is consistent with the results of XRD and Raman. Figure 2b shows the curve fitting data of the Sn 3d core-level spectra. Moreover, the Sn 3d doublet characterized by Sn 3d_3/2_–Sn 3d_5/2_ splitting peak can be clearly observed. The prominent peak of Sn 3d_5/2_ level is dissolved into two peaks centered at 486.77 and 486.15 eV, which can be attributed to Sn(IV) and Sn(II) configurations [26], respectively. The Sn 3d_3/2_ spectra exhibit two peaks at 495.14 and 494.51 eV, which are assigned to Sn^2+^ and Sn^4+^ [26]. As shown in Figure 2c, the binding energies (BE) of Ti 2p_3/2_ and Ti 2p_1/2_ are 458.5 and 464.2 eV respectively, which are ascribed to the Ti^4+^ oxidation states [27]. On the basis of the above discussion, it can be concluded that the TiO_2_/Sn_3_O_4_ sample is composed of Ti(IV), Sn(II and IV), and O, which is in good agreement with the XRD and Raman results. In addition, the calculated Ti/Sn ratio is 0.20, indicating that most of the surface of the TiO_2_ nanocrystals is covered by Sn_3_O_4_ nanocrystals (SEM and TEM experiments further confirm this result, and will be discussed later).

**Figure 3:**
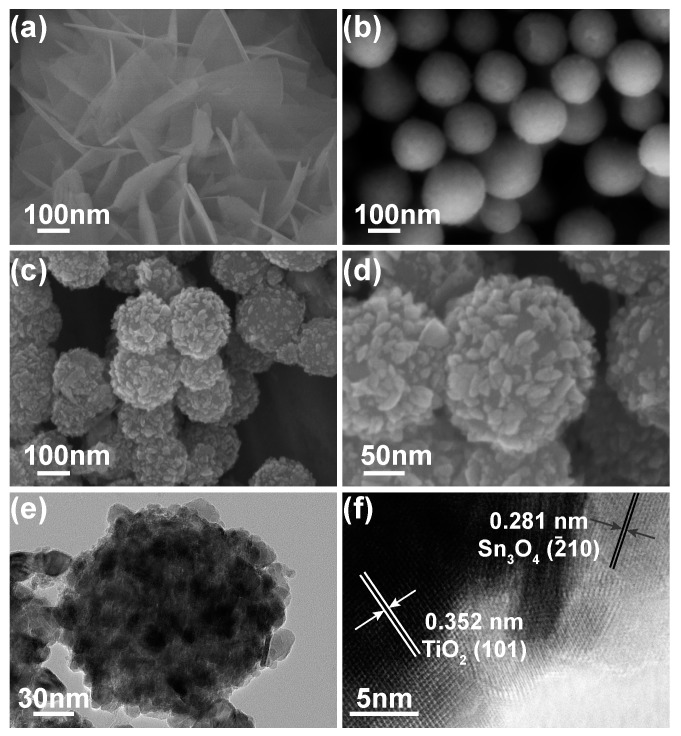
SEM images of the (**a**) Sn_3_O_4_, (**b**) TiO_2_, and (**c**,**d**) TiO_2_/Sn_3_O_4_ nanocomposites; (**e**) TEM and (**f**) HRTEM images of the TiO_2_/Sn_3_O_4_ nanocomposites.

The morphology and microstructure of the as-prepared TiO_2_/Sn_3_O_4_ nanocomposites were carefully analyzed by microscopy. Generally, lots of interleaved Sn_3_O_4_ nanoplates are able to self-assemble into an ordinary flower-like nanostructure, as shown in Figure 3a. Similarly, by introducing highly monodispersed TiO_2_ nanospheres of ~130 nm diameter (Figure 3b) into the growth environment, the homogeneous Sn_3_O_4_ nanoparticles started to grow on the surface of each individual TiO_2_ core with intimate contact, thus forming an interface of two different semiconductors that would facilitate photo-excited electron transfer and photon-generated carrier separation (Figure 3c,d). Noticeably, the composites inherit a favorable dispersion in the solution, and fully contact with the absorbate, which is positive for the outstanding photocatalytic performance. However, it should be noted that the TiO_2_/Sn_3_O_4_ nanocomposites are not completely covered by Sn_3_O_4_ nanocrystals. Furthermore, these advantageous heterojunctions with 10–20 nm sizes wrapping uniformly onto the surface of TiO_2_ nanospheres were verified by TEM observation, as shown in Figure 1e,f. The well-resolved lattice fringes from the core and shell regions manifestly correspond to the (101) planes of anatase TiO_2_ and the ($\bar{2}$10) planes of Sn_3_O_4_, respectively, clearly revealing the phase distribution of the TiO_2_/Sn_3_O_4_ nanocomposites again.

**Figure 4:**
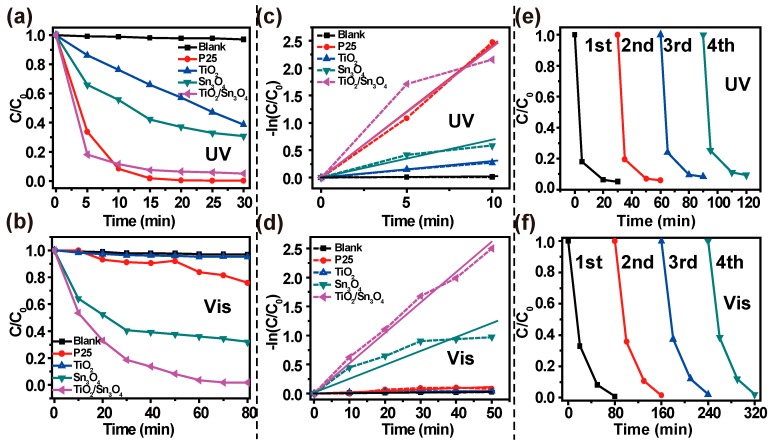
The photocatalytic activity (**a**,**b**), plots of -ln[C/C_0_] versus irradiation time (**c**,**d**), and stability for MO photo-degradation (**e**,**f**) of TiO_2_, P25, Sn_3_O_4_ and TiO_2_/Sn_3_O_4_ nanocomposites under UV- and visible-light irradiation, respectively. The corresponding curves of MO without photocatalyst under UV and visible light irradiation are provided for comparison.

The photocatalytic activities of P25, TiO_2_, Sn_3_O_4_ and TiO_2_/Sn_3_O_4_ heterostructures were evaluated by the degradation of MO in water under UV- and visible-light irradiation (Figure 4a,b).The degradation of the MO solution under identical experimental conditions, but with no photocatalyst, is provided for comparison. The degradation efficiency of the as-synthesized TiO_2_/Sn_3_O_4_ heterostructures was defined as C/C_0_, where C_0_ is the initial concentration of MO after equilibrium adsorption, and C is the concentration during the reaction. Both blank experiment results showed that MO could not be decomposed without photocatalyst under UV- or visible-light irradiation. In contrast, the photodegradation efficiency of TiO_2_/Sn_3_O_4_ nanocomposites was 95% within 30 min under UV-light irradiation, which is superior to the as-prepared TiO_2_ nanospheres (62%) and Sn_3_O_4_ nanoplates (70%). Furthermore, the MO decomposition efficiency found for the TiO_2_/Sn_3_O_4_ photocatalyst was comparable to that determined under the same experimental conditions for the reference P25 catalyst; that is, 99% after 10 min. Additionally, in the visible-light irradiation experiment (Figure 4b), the TiO_2_/Sn_3_O_4_ nanocomposites (81%) exhibited significantly higher photocatalytic activity than P25 (9%), TiO_2_ (0.4%) and Sn_3_O_4_ (60%) at 30 min. Finally, MO was completely degraded within 80 min. The results show that the TiO_2_/Sn_3_O_4_ heterostructures exhibited improved photocatalytic activity.

For a better understanding, the photocatalytic kinetics of the samples was analyzed using the Langmuir-Hinshelwood model, as shown in Figure 4c,d. All of the data follow a first-order reaction model, and the calculated apparent kinetic rate constants (κ) are summarized in Table 1. We found that, under UV irradiation, the TiO_2_/Sn_3_O_4_ exhibited a much faster photo-decomposition activity (κ = 0.24 min^−1^) than the pure TiO_2_ (0.028 min^−1^) and Sn_3_O_4_ (0.064 min^−1^), and was as fast as the P25 (0.24 min^−1^). Furthermore, under visible-light irradiation, the calculated value of κ for the TiO_2_/Sn_3_O_4_ sample (κ = 0.052 min^−1^) was twice as high as that for the neat Sn_3_O_4_ (0.024 min^−1^), and more than a dozen times higher than that for the single TiO_2_ (0.0010 min^−1^) and the P25 (κ = 0.0023 min^−1^). In addition, the TiO_2_/Sn_3_O_4_ heterostructures could be recycled and reused at least four times without significant loss of efficiency (Figure 4e,f), which demonstrates its great potential as an efficient and stable photocatalytic material. These remarkably good performances can be attributed to the improved UV- and visible-light absorption efficiency, and the high photo-excited carrier-separation rate resulting from the novel TiO_2_/Sn_3_O _4_ heterostructures.

**Scheme 1:**
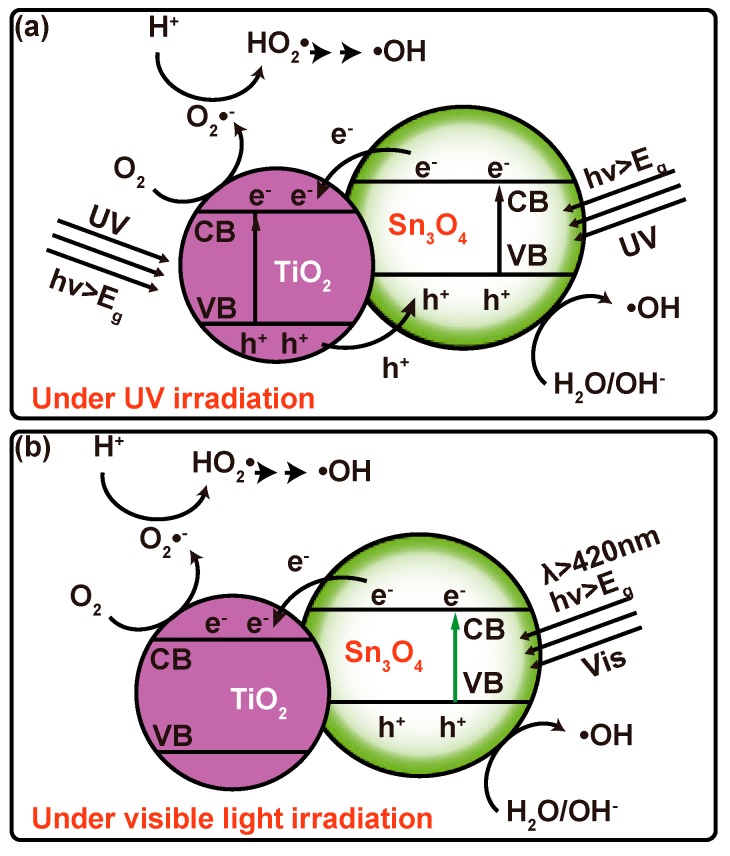
Illustration of photo-induced charge transfer and separation at the interface of TiO_2_/Sn_3_O_4_ hierarchical hybrid nanostructures under (**a**) UV- and (**b**) visible-light irradiation.

Based on all of the results above, a possible mechanism for charge transfer and photocatalytic process can be proposed (Scheme 1). As illustrated in Figure 1d, the diffusion reflectance spectra (DRS) and plots of (F(R)*hv*)^1/2^ versus photo energy (*hv*) indicate that the bandgap of Sn_3_O_4_ (2.61 eV) is smaller than that of TiO_2_ (3.22 eV). Additionally, the potentials of the valence band (VB) and conduction band (CB) of Sn_3_O_4_ are higher than those of TiO_2_, so the heterostructure of TiO_2_/Sn_3_O_4_ belongs to typical type-II heterojunction [9]. When Sn_3_O_4_ contacts TiO_2_ cores to form a heterojunction, the difference in chemical potential causes band bending at the interface of the junction [28], which drives photoexcited electrons to transfer from Sn_3_O_4_ to TiO_2_, and photoexcited holes to migrate in the opposite direction, until the Fermi levels of TiO_2_ and Sn_3_O_4_ reach equilibrium. The possible mechanisms for charge transfer and hydroxyl radical (•OH) generation under UV- and visible-light irradiation will be discussed separately. (1) Upon UV illumination, electrons in the VB could be excited to the CB of both oxides, simultaneously forming the same number of holes in the VB. This is due to the fact that the Sn_3_O_4_ nanoparticles were not fully coated as a shell onto the TiO_2_ nanospheres (Figure 3c,d) and that the suitable bandgap of TiO_2_ and Sn_3_O_4_ is lower than the energy of ultraviolet photons. Next, the photo-generated electrons were collected by the TiO_2_ particles and the holes by the Sn_3_O_4_ particles; that is, electrons transferred from Sn_3_O_4_ to TiO_2_, and holes migrated from TiO_2_ to Sn_3_O_4_ (compare Scheme 1a with Figure 1d). The unique behavior that electrons and holes preferentially accumulate on different materials would result in a great separation of photo-generated carriers, and thus reduce the charge recombination rate, ultimately increasing carrier lifetime. As a consequence, the formation efficiency of hydroxyl radicals (•OH)—a strong oxidant for most pollutants [9,20,29]—by the reaction of holes with surface hydroxyl groups or physisorbed water molecules at the Sn_3_O_4_ surface and the production rates of •OH and superoxide radicals (O_2_^−^) radicals resulting from the reactions of electrons with dissolved oxygen molecules and water molecules will be massively enhanced; this will increase the volume of oxidant inside the system. (2) Under visible-light irradiation (Scheme 1b), electrons in the VB could be exclusively excited to the CB of Sn_3_O_4_, with a concomitant formation of the same number of holes in the VB. Due to the type II band alignment of the as-prepared sample, the photoexcited electrons in the Sn_3_O_4_ CB will be easily injected into the TiO_2_ CB, where the electrons could reduce surface-absorbed O_2_ over TiO_2_ active sites to form superoxide radicals (O_2_^−^), and the new species can further yield •OH by reacting with water or oxidize MO. On the other hand, holes remaining in Sn_3_O_4_ could react with surface-absorbed H_2_O to generate more •OH. Hydroxyl radicals (•OH) and superoxide radicals (O_2_^−^) stemming from the above procedure will degrade MO into colorless chemicals, and even CO_2_ and H_2_O, which is similar under UV illumination. All in all, the enhanced charge separation related to the TiO_2_/Sn_3_O_4_ heterojunction favors the interfacial charge transfer to physisorbed species, forming •OH radicals and reducing possible back reactions, and therefore accounts for the higher activity of the TiO_2_/Sn_3_O_4_ nanocomposites.

**Figure 5:**
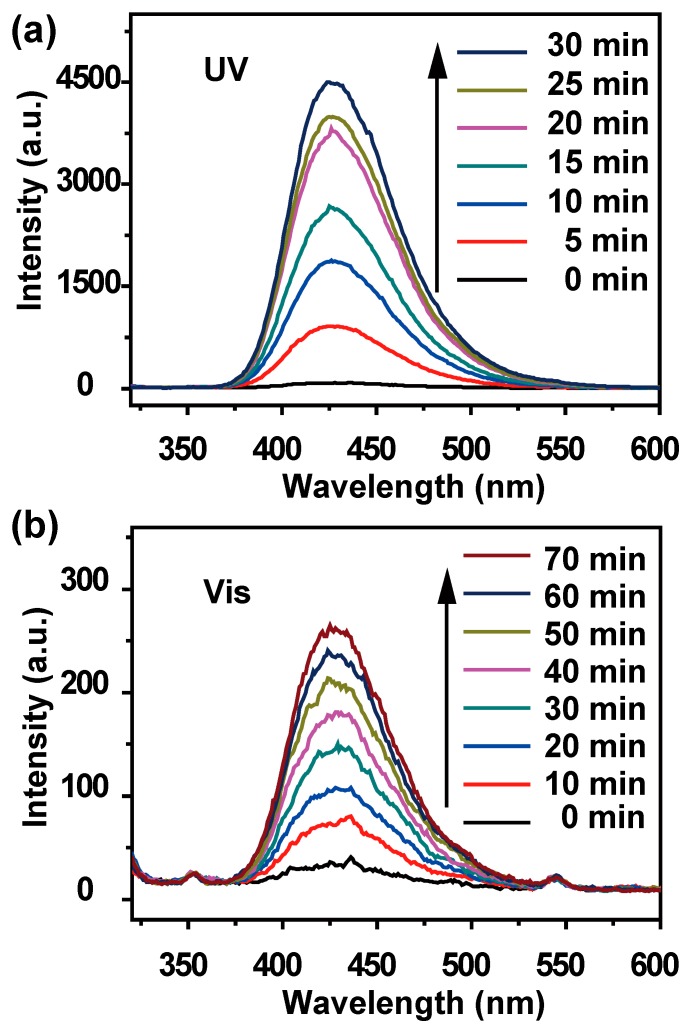
The •OH-trapping photoluminescence spectra of TiO_2_/Sn_3_O_4_ nanocomposites under (**a**) UV- and (**b**) visible-light irradiation, respectively.

The photocatalytic oxidation of dyes occurs through the reactive species, which came into being after the light absorption and electron-hole formation by the photocatalyst [30]. Terephthalic acid photoluminescence probing technique (TAPL) was employed to examine the generation of active •OH radicals [31]. Figure 5a,b gives the •OH-trapping photoluminescent spectra of TiO_2_/Sn_3_O_4_ nanocomposites in TA solution with UV- and visible-light irradiation, respectively. The increased photoluminescence intensity confirms that the •OH radicals are mainly responsible for the photodegradation process, and it also verifies the photocatalytic activity of the TiO_2_/Sn_3_O_4_ nanocomposites.

## 4. Conclusions

In summary, this study demonstrates a facile route to synthesizing TiO_2_/Sn_3_O_4_ nanocomposites that not only display enhanced photocatalytic performance in UV irradiation, but also allow a significant level of visible light photocatalytic activity. The large surface area derived from the monodispersed mesopore TiO_2_/Sn_3_O_4_ nanospheres and unique TiO_2_/Sn_3_O_4_ heterojunctions are considered to be major contributions to supplying abundant active sites and separating photogenerated carriers, respectively. The strengthened photocatalytic performances will greatly promote the practical application of the TiO_2_/Sn_3_O_4_ nanocomposites in eliminating organic pollutants from wastewater, and producing hydrogen by splitting.

## Figures and Tables

**Table 1:** The specific surface area and apparent reaction rate constants (κ) of TiO_2_, P25, Sn_3_O_4_ TiO_2_/Sn_3_O_4_ samples.

Photocatalyst	TiO_2_	P25	Sn_3_O_4_	TiO_2_/Sn_3_O_4_
	κ (min^−1^)	κ (min^−1^)	κ (min^−1^)	κ (min^−1^)
UV irradiation	0.028	0.24	0.064	0.24
Visible light	0.0010	0.0023	0.024	0.052
Surface Area (m^2^∙g^−1^)	0.04	50	35.2	68.1
